# High-intensity interval training: optimizing oxygen consumption and time to exhaustion taking advantage of the exponential reconstitution behaviour of D’

**DOI:** 10.1007/s00421-022-05059-2

**Published:** 2022-10-15

**Authors:** Filippo Vaccari, Jacopo Stafuzza, Nicola Giovanelli, Stefano Lazzer

**Affiliations:** 1 Department of Medicine, University of Udine, P.le Kolbe 4, 33100 Udine, Italy; 2 School of Sport Sciences, University of Udine, Udine, Italy

**Keywords:** D’, Critical velocity, Time at ${\dot{\text{V}}\text{O}}_{{{\text{2max}}}}$, HIIT protocols, D’ recovery, Reconstruction, HIDIT

## Abstract

**Purpose:**

Accumulating the time near maximum aerobic power $\left( {{\dot{\text{V}}\text{O}}_{{{\text{2max}}}} } \right)$ is considered to be the most effective way to improve aerobic capacity. The aims of this study were: (1) to verify whether postponing the first recovery interval improves time to exhaustion during a high-intensity interval training (HIIT) test, and (2) to verify whether *a HIIT* protocol with decreasing interval duration (HIDIT) is more effective in accumulating time near ${\dot{\text{V}}\text{O}}_{{{\text{2max}}}}$ compared with two classical protocols with short intervals (SI_HIIT_) and long intervals (LI_HIIT_).

**Methods:**

Nine active males (35 ± 11 years, ${\dot{\text{V}}\text{O}}_{{{\text{2max}}}}$ 52 ± 5 mL·min^−1^·kg^−1^) performed a graded exercise test on an athletic track. Critical velocity and D’ were estimated from three to five high-intensity trials to exhaustion. Then, the subjects performed three trials with a single recovery interval after 30 s (Rec_30s_), after 3 min (Rec_3min_) and after exhaustion (Rec_Tlim_) to verify whether postponing the first recovery interval enhances the time to exhaustion. Finally, the subjects performed the three HIIT protocols mentioned above.

**Results:**

The time to exhaustion was significantly greater in Rec_Tlim_ (464 ± 67 s) than in Rec_3min_ (388 ± 48 s) (*p* < 0.0078) and Rec_30s_ (308 ± 44 s) (*p* > 0.0001). Additionally, it was significantly greater in Rec_3min_ than in Rec_30s_ (*p* = 0.0247). Furthermore, the time accumulated near ${\dot{\text{V}}\text{O}}_{{{\text{2max}}}}$ was significantly longer in HIDIT (998 ± 129 s) than in SI_HIIT_ (678 ± 116 s) (*p* = 0.003) and LI_HIIT_ (673 ± 115 s) (*p* < 0.031).

**Conclusions:**

During the trials, postponing the first recovery interval was effective in improving the time to exhaustion. Moreover, HIDIT was effective in prolonging the time near ${\dot{\text{V}}\text{O}}_{{{\text{2max}}}}$.

## Introduction

Maximum aerobic power (${\dot{\text{V}}\text{O}}_{{{\text{2max}}}}$) is one of the most important parameters for evaluating the efficiency of the cardiorespiratory system, and it is also one of the most important determinants of endurance performance. Therefore, the purpose of many training protocols is to improve maximum aerobic power by working at intensities close to or equal to ${\dot{\text{V}}\text{O}}_{{{\text{2max}}}}$ (Wenger and Bell 1986; Midgley and Mc Naughton 2006). Researchers have proposed different protocols that were able to solicit and maintain the exercise intensity close to ${\dot{\text{V}}\text{O}}_{{{\text{2max}}}}$ as long as possible. One of the training methods that allows athletes to extend the duration of exercise at a very high intensity is called high-intensity interval training (HIIT) (Midgley and Mc Naughton 2006; Buchheit and Laursen 2013). HIIT can be set with the help of critical velocity (CV) and D’ (maximum distance that may be covered beyond CV (Jones et al. 2019)). CV is the intensity below which a metabolic steady state is still possible (for a more detailed definition see Jones and Vanhatalo (2017)). CV and D′ are phenomenologically equivalent to critical power (the critical threshold used in performances where the intensity is measured in Watts such as cycling) and W′ (the finite amount of work that can be done beyond CP), respectively (Ettema 1966; Broxterman et al. 2015; Jones and Vanhatalo 2017; Jones et al. 2019). During HIIT, D' is consumed when the intensity is above CV. On the other hand, during recovery periods below CV, D’ is reconstituted (Billat et al. 2001; Skiba et al. 2014; Jones and Vanhatalo 2017). When D' is fully depleted, only an adequate recovery period allows to perform another high-intensity interval. Nevertheless, while the depletion of D’ above CV occurs at a constant rate, its reconstitution takes place with exponential behaviour. In other words, whether D' is close to exhaustion, its recovery is quicker than when it is about to be fully reconstituted. In fact, the rate of recovery decreases as a function of recovery time (Ferguson et al. 2010; Skiba et al. 2014; Caen et al. 2019; Sreedhara et al. 2020; do Nascimento Salvador et al. 2021; Lievens et al. 2021).

In a previous work (Vaccari et al. 2020) we showed that this feature can be exploited to extend the time elapsed above 90% of ${\dot{\text{V}}\text{O}}_{{{\text{2max}}}}$ (T90%${\dot{\text{V}}\text{O}}_{{{\text{2max}}}}$) during HIIT. HIIT protocols with initially longer and then shorter intervals above CV would allow prolongation of T90%${\dot{\text{V}}\text{O}}_{{{\text{2max}}}}$ for two reasons: 1—an initial "priming effect" of oxygen consumption (Jones et al. 2008): indeed, long intervals increase oxygen consumption faster than short intervals (Millet et al. 2003); 2—at the beginning of the exercise, when D' is not widely depleted, its recovery is relatively slow (Skiba et al. 2012). In contrast, the reconstitution of D' will be relatively faster starting with a long interval and thus recovering when D’ is already nearly depleted (Skiba et al. 2012). After the first long interval, more frequent recovery periods could be introduced by decreasing the duration of the intervals. In fact, at this stage, even a few seconds of recovery will be more effective than at the beginning. This should allow for the extension of high-intensity exercises for longer and maintain the rate of oxygen consumption high for longer time.

The current study aims to expand our previous work (Vaccari et al. 2020) and tests the hypothesis that: (1) comparing three trials with a single recovery period, postponing the first recovery interval recovery allows to prolong the time to exhaustion at the end of the trial; (2) decreasing the intervals duration during a HIIT session can be more effective in running performance in comparison with HIIT protocol with long or short intervals.

## Materials and methods

### Subjects

Nine active, non-smoking men were recruited (34 ± 11 years; 79 ± 8 kg; ${\dot{\text{V}}\text{O}}_{{{\text{2max}}}}$ 52 ± 5 mL·min^−1^·kg^−1^). All of them concluded the entire protocol except one who got injured in the last test. Then, all of his completed tests were taken into account for the analysis. The inclusion criteria were: (1) recreational runner; (2) training volume in the latest 6 months was greater than 3 session per week (or was greater than 40 km per week … vedi tu); (3) none of the athletes had a history of neuromuscular or musculoskeletal impairments at the time of the study that could affect the results.

### Study design

**Fig. 1:**
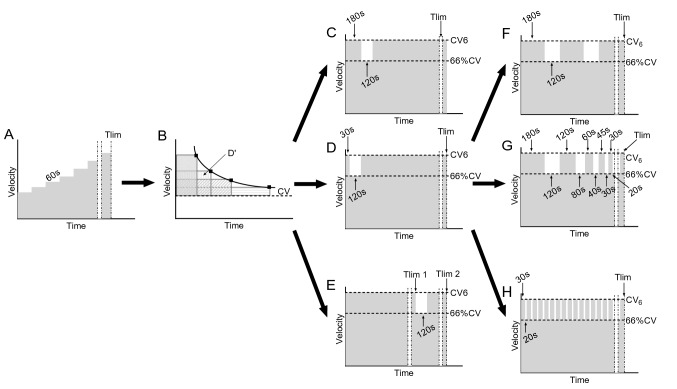
Study design. All participants performed an incremental test (**A**). Then, they performed three to five high-intensity trials to estimate their velocity–duration relationship (**B**), used to prescribe the intensities of all the following sessions. The high intensity was set as the velocity that was supposed to lead to exhaustion in 6 min (CV_6_), while low intensity was the velocity corresponding to the 66% of critical velocity (66%CV). The tests **C**, **D** and **E** were used to measure how much the timing of the first recovery bout [after 30 s, (**C**); 3 min, (**D**); or Tlim, (**E**)] influences the reconstruction rate of D’. Finally, the subjects performed the three HIIT protocols: Long intervals HIIT (LI_HIIT_), (**F**); high-intensity decremented interval training (HIDIT), (**G**); Short intervals (SI_HIIT_), (**H**)

The Ethics Committee of the Friuli-Venezia Giulia approved the study (protocol number 30/2021). Participants performed from 9 to 11 test days, separated by at least 48 h in the spring of 2021. During the first visit to the laboratory, an operator explained the purposes and the objectives of the study to each subject and obtained written informed consent. Then, participants underwent medical examinations and performed a maximal running step-incremental test to measure ${\dot{\text{V}}\text{O}}_{{{\text{2max}}}}$ and maximal aerobic speed (${\text{v}}{\dot{\text{V}}\text{O}}_{{{\text{2max}}}}$). Although the objectives were explained to all subjects, the study hypothesis was not revealed to not influence the results. After the first visit, participants were examined three to five times on different days to determine their velocity–duration relationship (CV and D’). CV parameters were used to set up the HIIT tests. Then, they randomly performed three tests to exhaustion to determine the reconstitution of D’ after a period of 2 min of recovery, which was set after high-intensity bouts of 30 s, 3 min or after Tlim (time to exhaustion). Finally, the subjects performed three HIIT tests in a randomized order: long interval HIIT (LI_HIIT_), high-intensity decreasing interval training (HIDIT), and short interval HIIT (SI_HIIT_) (Fig. 1). During the three HIIT tests, oxygen consumption and heart rate (HR) were measured along with Tlim, and the time above 90% of ${\dot{\text{V}}\text{O}}_{{{\text{2max}}}}$ and blood lactate concentration (BLC), HR and rate of perceived exertion (RPE) using the Borg CR-10 scale (Borg et al. 2010) were measured at the end of the tests. Each test was performed at the same time of the day, on an athletic track and separated from the previous one by a minimum of 2 and a maximum of 6 days. Subjects were instructed to avoid the consumption of drinks containing caffeine for at least 8 h before each test and to avoid vigorous physical activity in the 24 h preceding each testing session. Furthermore, they were asked to maintain the same drinking and feeding habits before each test. The nine subjects concluded the entire protocol within 12 weeks from the first visit.

### On-track testing session

Incremental tests, velocity–duration relationship trials, D’ reconstitution trials, and HIIT tests were performed at the athletic track of Gemona del Friuli, Italy. Every test session was preceded by the same warm-up routine: 10 min running at 10 or 12 km/h (depending on the fitness level), followed by 1-min passive recovery. The running speed was set considering a series of markers that were placed every 20 m on the first lane of the track. Every participant was paced by an operator on a bike that set the right speed passing every mark when an acoustic signal (a beep) was emitted from a device. In other words, each "beep" corresponded to the time in which 20 m should have been covered if one went at the predetermined speed (e.g., at 2.7 m/s a beep every 7.4 s). The test ended when the subject was not able to follow the bike for two consecutive markers, despite strong encouragement of the operator.

### Incremental test

The incremental test was performed on the athletic track under medical supervision, and standard safety procedures were followed. During the first visit, an operator instructed the subjects to correctly report the rate of perceived exertion on the CR-10 scale (Borg et al. 2010). The incremental step test started at 10 or 12 km/h depending on the fitness level of the subject and increased by 0.5 km/h per minute throughout the test until voluntary exhaustion. Ventilation, ${\dot{\text{V}}\text{O}}_{{2}}$ and carbon dioxide (${\dot{\text{V}}\text{CO}}_{{2}}$) were measured breath-by-breath using a portable metabolic unit (K5, Cosmed, Italy). The ventilation was measured by a turbine calibrated before each test with a 3-L syringe. Calibration of O_2_ and CO_2_ analysers was performed before each test by utilizing calibration gas mixtures of known composition (16.00% O_2_; 4.00% CO_2_). The V̇O_2_ plateau during the last 30 s was used to verify the achievement of ${\dot{\text{V}}\text{O}}_{{{\text{2max}}}}$, while ${\text{v}}{\dot{\text{V}}\text{O}}_{{{\text{2max}}}}$ was considered the minimum velocity at which the ${\dot{\text{V}}\text{O}}_{{{\text{2max}}}}$ plateau appeared (Taylor et al. 1955; Poole and Jones 2017).

### Velocity–duration relationship

CV and D’ were estimated from three to five high-intensity trials to exhaustion performed in different days at 90 to 110% of the ${\text{v}}{\dot{\text{V}}\text{O}}_{{{\text{2max}}}}$. All the tests had to last a minimum of ~ 2 and a maximum of ~ 15 min to be considered valid (Jones and Vanhatalo 2017).

To find CV and D’, two methods were used (Clarke and Skiba 2013): (1) Distance–time method: the distance covered (D) in each of the separate exercise bouts was plotted against T_lim_. In this case, the slope of the linear regression represents CV, and the intercept represents D’; (2) 1/time method: velocity was plotted against 1/time. In this case, the slope of the line represents D’, and the intercept represents CV. For each subject, the determination coefficient (*R*^2^) resulting from both methods had to be higher than 0.95. If not, more trials were performed to increase the precision of linear regression. A minimum of three and a maximum of five trials have been made for each subject. Eventually, the method with higher *R*^2^ was used to determine CV and D’.

### D’ reconstitution tests

To investigate the reconstruction characteristics of D’, the participants performed three trials in a randomized order and preceded by the same warm-up as in the incremental test. The three trials comprised one high-intensity bout (B1) followed by a 2-min active recovery interval and a second high-intensity bout to exhaustion (B2). The first bout of the trial was meant to simulate three different interval durations in which D’ was consumed: long bout (Rec_3min_, Fig. 1C), short bout (Rec_30sec_, Fig. 1D) and exhaustion bout (Rec_Tlim_, Fig. 1E). The intensity of the high-intensity bouts was the same as the HIIT test and corresponded to the velocity that was supposed to lead to exhaustion in 6 min (360 s) according to the following equation (Jones et al. 2010):

1 $${\text{CV}}_{{6}} = \frac{{D^{\prime}}}{360s} + CV.$$

The velocity used for the low-intensity bout was 66% of the CV and was the same as that used in the HIIT test.

The recovery interval of 2 min served to the reconstitution of D’ (Caen et al. 2019), and the last bout to exhaustion was performed to measure how much the timing of the first recovery bout (after 30 s, 3 min or Tlim) influenced the reconstitution rate of D’. Because it can be theoretically assumed that D′ = 0 at exhaustion, this calculation yielded the D′ reconstitution after the recovery interval.

The amount of D′ expended in the first bout (B1) and in the second bout (B2) (Fig. 1C–E) was calculated by numerical integration of the power values above CV. The difference between D’ was calculated from the intensity–duration relationship, and the sum of the expenditure of D’ during B1 and B2 allowed us to calculate the amount of D' reconstituted during the recovery interval. Subsequently, it was expressed as a percentage of total D’.

### HIIT tests

After the incremental tests, the critical velocity tests, and the recovery trials, subjects performed three HIIT training sessions in a randomized order. The velocity of the work/recovery bouts and the work/recovery duration ratio were the same in each trial, although the duration of the intervals was changed. The work and recovery intensity were the same as the D’ reconstitution tests (see previous paragraph), while the ratio work/recovery time was set at 3/2 for all the tests. The three-interval test was structured as follows:

- Long intervals (LI_HIIT_, Fig. 1F): 3 min at high intensity and 2 min at low intensity repeated until volitional exhaustion of the subject;
- High-intensity decremental interval training (HIDIT, Fig. 1G): 3 min at high intensity and 2 min at low intensity; 2 min at high intensity and 1 min and 20 s at low intensity; 1 min at high intensity and 40 s at low intensity; 45 s at high intensity and 30 s at low intensity; and finally, 30 s at high intensity and 20 s at low intensity repeated until volitional exhaustion of the subject;
- Short intervals (SI_HIIT_, Fig. 1H): 30 s at high intensity and 20 s at low intensity until volitional exhaustion of the subject.

Throughout the HIIT protocols, ventilatory parameters were measured using a breath-by-breath metabolic unit (K5, Cosmed, Italy), and then data were averaged every 5 s. ${\dot{\text{V}}\text{O}}_{{2}}$ and HR were measured during the entire test, while BLC and RPE were measured at the end of each test. An operator collected a capillary blood sample from the fingertip to measure BLC after 3 min of the end of the exercise (Lactate Scout 4, EKF Diagnostics, UK), while the subjects reported RPE consulting the CR-10 scale shown by another operator. At the end, the total time spent above 90% of ${\dot{\text{V}}\text{O}}_{{{\text{2max}}}}$ was determined as the sum of each averaged 5 s when ${\dot{\text{V}}\text{O}}_{{2}}$ was equal to or higher than 90% of ${\dot{\text{V}}\text{O}}_{{{\text{2max}}}}$.

### Statistics

Statistical analysis was performed using GraphPad Prism 8.0.2 software (IBM, Chicago, USA) with significance set at *P* < 0.05. Descriptive data are presented as the mean ± SD. HIIT tests and the three sessions to test D’ reconstitution were compared by a mixed-effect analysis. Where the analysis found a significant difference, Tukey’s multiple comparison test between the three protocols was performed. Effect size (ES) was calculated using Cohen’s *d* (0 < *d* < 0.20, *small*; 0.20 < *d* < 0.50, *medium*; *d* > 0.50, *large*). For our purposes, a sample size of 6 subjects was calculated to have a statistical power of 0.8 to refute the null hypothesis and to obtain an ES of 0.80 with an alpha error of 0.05 and a beta error of 0.20. To have a safe margin 9 subject have been recruited.

## Results

Age and weights along with values attained during the incremental test and the velocity–duration relationship trials are shown in Table 1.

**Table 1:** Anthropometrics and physiological characteristic of the participants; data were obtained from incremental test and velocity–duration relationship trials

Subject	Age (yr)	Weight (kg)	${\dot{\text{V}}\text{O}}_{{{\text{2max}}}}$ (mLO_2_ × min^−1^)	${\dot{\text{V}}\text{O}}_{{{\text{2max}}}}$ (mLO_2_ × kg^−1^ × min^−1^)	${\rm{v}} \dot {{\rm V}}{{\rm{O}}}_{2{\rm max}}$ (km × min^−1^)	HR_max_ (bpm)	CV (m × s^−1^)	D' (m)
1	29	86	4125	48.0	15.0	186	3.48	250
2	21	69	3801	55.1	18.0	181	4.39	248
3	44	86	4660	54.2	15.0	175	3.47	204
4	44	85	3807	44.8	14.0	191	3.34	246
5	21	88	4356	49.5	14.0	194	3.37	230
6	42	77	3510	45.6	13.5	165	3.33	121
7	51	78	3990	51.2	14.5	164	3.40	163
8	36	71	4310	60.7	17.5	149	4.48	124
9	28	68	3866	56.9	15.5	183	3.78	258
Mean	35	79	4047	52	15.2	176	3.67	205
SD	11	8	352	5	1.6	15	0.45	55

${\dot{\text{V}}\text{O}}_{{{\text{2max}}}}$: maximal aerobic power; ${\mathrm{v\dot V}}{{\mathrm{O}}_{{\mathrm{2max}}}}$: velocity at ${\dot{\text{V}}\text{O}}_{{{\text{2max}}}}$; ${\text{HR}}_{{{\text{max}}}}$: maximal heart rate; CV: critical velocity; D’: amount of work which is possible to do beyond CV expressed as a distance in metres

**Fig. 2:**
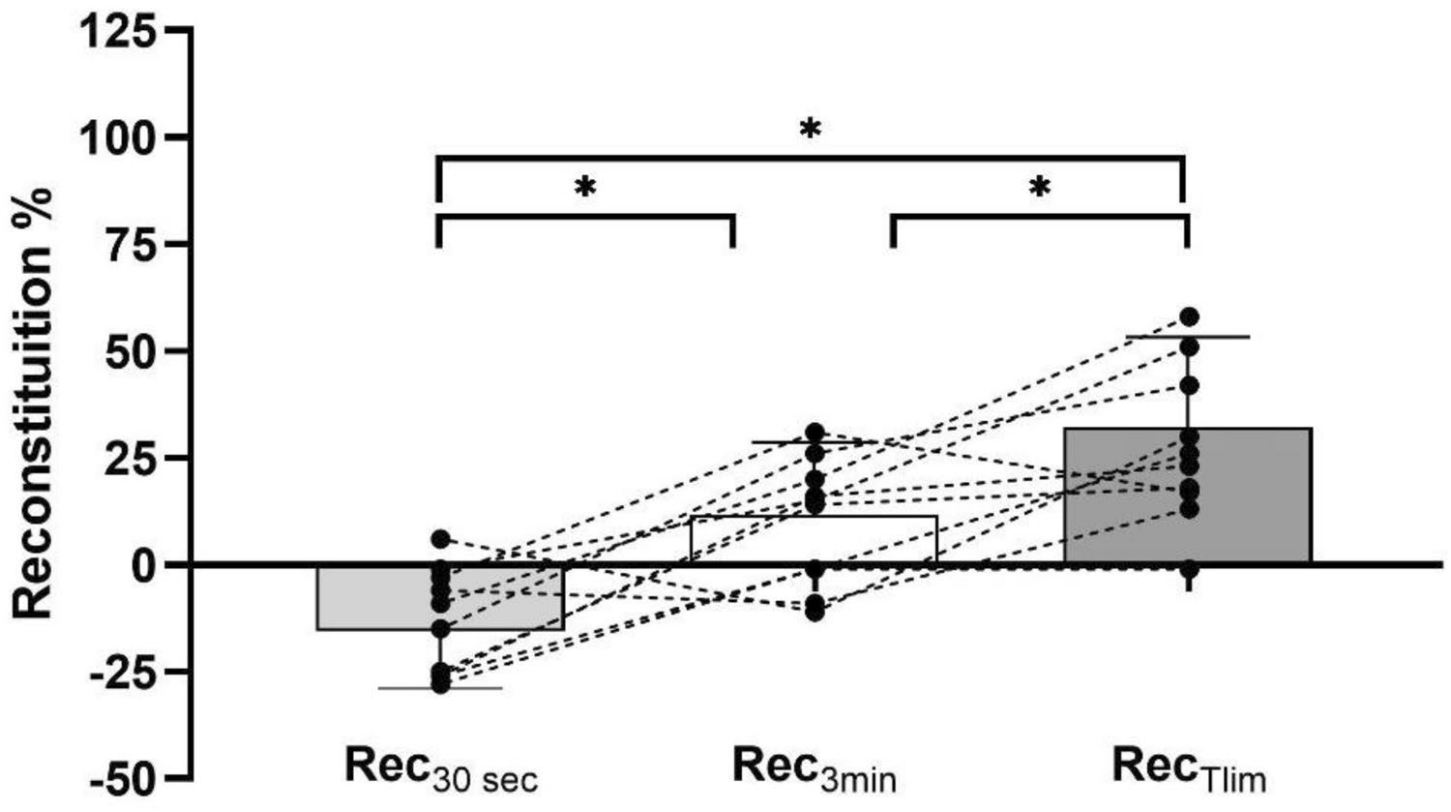
Reconstitution as a percentage of D’ during 2 min of recovery at velocity corresponding to 66% of CV, timed after 30 s (Rec_30sec_), 3 min (Rec_3min_) and after exhaustions (Rec_Tlim_) at the intensity of CV_6_ (the velocity that was supposed to lead to exhaustion in 6 min). *significantly different (*P* < 0.05)

For trials investigating D’ and for HIIT protocols, the high intensity was set at CV_6_ (mean value 15.2 ± 1.6 km/h), while low intensity corresponded to 66% CV (mean value 8.8 ± 1.6 km/h). The total time spent at high intensity during the reconstitution D' trials was on average 308 ± 44 s, 388 ± 48 s, and 464 ± 67 s for Rec_30sec_, Rec_3min_, and Rec_Tlim_, respectively, which was significantly different between each trial (Fig. 2). In particular, Rec_3min_ was greater than Rec_30sec_ (+ 80 s; 95% C.I from + 150 to + 12 s; *p* = 0.0247; ES = 1.00, *large*); Rec_Tlim_ was greater than Rec_30sec_ (+ 156 s; 95% C.I from + 213 to + 100 s; *p* > 0.0001; ES = 2.64, *large*); and Rec_Tlim_ was greater than Rec_3min_ (+ 75 s; 95% C.I from + 128 to + 24 s; *p* = 0.0078; ES = 1.39, *large*).

Furthermore, D' of Rec_30sec_ was on average 13 ± 12% less than D' calculated from the velocity–duration relationship, while following Rec_3min,_ D' was + 10 ± 15%, and following Rec_Tlim,_ D' was + 28 ± 18% (Fig. 2).

**Fig. 3:**
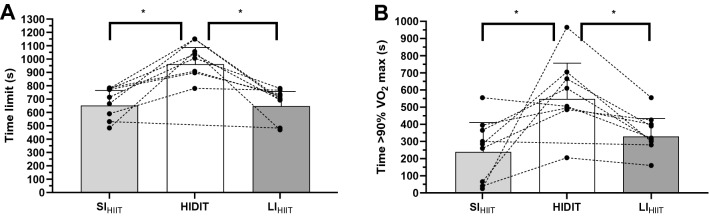
Time > 90% ${\dot{\text{V}}\text{O}}_{{{\text{2max}}}}$ (**A**) and Tlim (**B**) during HIIT protocols with short intervals (SI_HIIT_), with decreasing duration of intervals (HIDIT) and with long intervals (LI_HIIT_) with same mean speed as high intensity, low intensity and the ratio of the durations of high/low intensities are identical. *Significantly different (*P* < 0.05)

The physiological and perceptual responses during the three HIIT protocols are shown in Table 2. There were no significant differences between the three protocols other than T_lim_ and T90%${\dot{\text{V}}\text{O}}_{{{\text{2max}}}}$. In particular, the T_lim_ of HIDIT was significantly higher than those of SI_HIIT_ (+ 48%; 95% C.I from + 488 to + 154 s; *p* = 0.001; ES = 1.85, *large*) and LI_HIIT_ (+ 47%; 95% C.I from + 519 to + 133 s; *p* = 0.004; ES = 1.60, *large*), while there were no differences between the T_lim_ of SI_HIIT_ and LI_HIIT_ (Fig. 3B). Furthermore, T90%${\dot{\text{V}}\text{O}}_{{{\text{2max}}}}$ of HIDIT was significantly higher than SI_HIIT_ (+ 116%; 95% C.I from + 440 to + 20 s; *p* = 0.003; ES = 1.04, *large*) and LI_HIIT_ (+ 66%; 95% C.I from + 535 to + 115 s, *p* = 0.031; ES = 1.50; *large*), while there were no differences between T90%${\dot{\text{V}}\text{O}}_{{{\text{2max}}}}$ of SI_HIIT_ and LI_HIIT_ (Fig. 3A).

**Table 2:** Data from HIIT protocol with short intervals (SI_HIIT_), with decreasing duration of intervals (HIDIT) and with long intervals (LI_HIIT_)

	SI_HIIT_	HIDIT	LI_HIIT_
Tlim (s)	678 ± 116*	998 ± 129	673 ± 115*
${\dot{\text{V}}\text{O}}_{{{\text{2peak}}}}$(mL O_2_ × min^−1^)	3890 ± 442	4032 ± 393	4277 ± 388
% ${\dot{\text{V}}\text{O}}_{{{\text{2peak}}}}$/${\dot{\text{V}}\text{O}}_{{{\text{2max}}}}$	96 ± 9	101 ± 6	106 ± 9
${\dot{\text{V}}\text{O}}_{{{\text{2mean}}}}$(mL O_2_ × min^−1^)	3590 ± 397	3580 ± 292	3637 ± 306
% *V̇*O_2mean_/*V̇*O_2max_	89 ± 6	89 ± 5	90 ± 8
T90%${\dot{\text{V}}\text{O}}_{{{\text{2max}}}}$(s)	167 ± 188*	579 ± 219	349 ± 111*
HR_peak_ (bpm)	175 ± 15	176 ± 13	176 ± 11
HR_mean_ (bpm)	166 ± 16	164 ± 15	164 ± 16
BLC post (mM)	7.5 ± 4.7	9.9 ± 4.5	8.6 ± 3.0
RPE	8.2 ± 0.7	8.5 ± 0.4	8.3 ± 0.4

For all the protocols the same mean speed for high intensity and low intensity were used, and the ratio of the durations of high/low intensities were identical

Values are presented as mean ± SD

Tlim: time to exhaustion; ${\dot{\text{V}}\text{O}}_{{{\text{2peak}}}}$: peak oxygen consumption during the HIIT protocols; ${\dot{\text{V}}\text{O}}_{{{\text{2mean}}}}$: mean oxygen consumption during the HIIT protocols; ${\dot{\text{V}}\text{O}}_{{{\text{2max}}}}$: maximal oxygen consumption from the incremental test; T90%${\dot{\text{V}}\text{O}}_{{{\text{2max}}}}$: Time elapsed above 90% of ${\dot{\text{V}}\text{O}}_{{{\text{2max}}}}$; ${\text{HR}}_{{{\text{peak}}}}$: peak heart rate; ${\text{HR}}_{{{\text{mean}}}}$: mean heart rate during the work intervals; BLC post: blood lactate concentration 3 min after the end of the protocol; RPE: rate of perceived exertion. *different from HIDIT (*p* < 0.05)

## Discussion

The main findings of the present study showed that (i) postponing the first recovery interval allows increasing T_lim_. In fact, Rec_tlim_ was more effective than Rec_3min_ and Rec_30sec_, and Rec_3min_ was more effective than Rec_30sec_ in prolonging time to exhaustion; (ii) time above 90% of ${\dot{\text{V}}\text{O}}_{{{\text{2max}}}}$ and T_lim_ were higher during HIDIT than HIIT protocols with long (LI_HIIT_) and short (SI_HIIT_) intervals, despite having identical mean velocity, duration and ratio high/low intensity of the intervals. These results confirm that the reconstitution of D’, when falling below the intensity corresponding to the CV, has an exponential behaviour that can be exploited in HIIT protocols to prolong T_lim_ and T90%${\dot{\text{V}}\text{O}}_{{{\text{2max}}}}$.

The intensities used for the HIIT protocols were chosen to optimize the time near to maximum oxygen consumption. The velocity used for the high-intensity intervals (CV_6_), corresponds approximately to the speed at ${\dot{\text{V}}\text{O}}_{{{\text{2max}}}}$ (Billat et al. 1999). The velocity used for the low intensity intervals (66%CV), is slow enough to allow recovery, but fast enough to maintain high the oxygen consumption. The choice of protocols for HIIT with long and short intervals was also made to optimize the time near to maximum oxygen consumption, as emerged from the indications of the review of Buchheit and Laursen (2013). The HIDIT protocol, on the other hand, was designed to progressively arrive from long to short intervals over a not excessively long time.

Several studies performed on cycle ergometers have verified that the reconstitution of W' (used in place of D’ when cycling) was exponential (Ferguson et al. 2010; Skiba et al. 2014; Caen et al. 2019; Sreedhara et al. 2020; do Nascimento Salvador et al. 2021; Lievens et al. 2021), while the present study is the first to verify it on running performance.

Furthermore, our study used a different experimental design from previous studies. For example, the studies by Ferguson et al. (2010) and Caen et al. (2019) demonstrated that the reconstitution of W' during recovery was exponential by performing a series of tests with recovery intervals with progressively longer durations. Thus, they verified that after reaching exhaustion, at the beginning, the reconstitution of W’ was very fast and then gradually slowed down. In recent years, this topic has been of interest to several studies that have tried to better explain the behaviour of W', both during bouts above critical power (CP, used in place of CV when cycling) and below CP. The studies by Skiba and colleagues (Skiba et al. 2012, 2014, 2015; Chidnok et al. 2013; Broxterman et al. 2015) led to the creation of a model that allows us to predict the behaviour of W' (i.e., the Model W' bal). Recent studies have tried to question it. For example, the study by Chorley et al. (2018) showed that repeated exhaustion exercises slow down the reconstruction of W’. The study by Caen et al. (2019) showed that the speed of reconstitution of W' depends on how quickly it was depleted by the previous bout: the faster it is consumed, the faster it rebuilds. Furthermore, the study by Lievens et al. (2021) showed how the reconstitution of W' was slower than expected in the *heavy* domain. Finally, the study by Sreedhara et al. (2020) showed that the *intensity* of recovery was even more important than the *duration* of recovery. In agreement with previous studies on cycling, our study confirmed the exponential trend of the reconstitution of D’ during running performance. Moreover, it showed that this feature can be exploited to increase exercise tolerance by postponing the first recovery interval.

Unexpectedly, in the Rec_30sec_ test, the total time over CV was even less than that estimated by the velocity–duration relationship tests (Fig. 2). This means that the 2 min under CV did not allow for recovery but probably even consumed a portion of D'. Unfortunately, we cannot explain this result through our data, and there are no other studies that have verified the behaviour of W' (cycling) or D' (running) after such a short interval above CV. A possible explanation is that the first 30 s were not sufficient to activate the aerobic system adequately. Therefore, the recovery carried out in the following 2 min (i.e., at 66% of the CV) was not sufficiently low to allow D’ reconstitution. Indeed, the study by Sreedhara et al. (2020) showed that at an intensity close to CP, the reconstitution of W' is much slower than one might expect, even at approximately 90% CP intensity. In some cases, a depletion of W’ occurs instead of a reconstitution.

We previously observed that a HIDIT protocol can increase the T90%${\dot{\text{V}}\text{O}}_{{{\text{2max}}}}$ in cyclists compared to HIIT protocols with short or long intervals (Vaccari et al. 2020). However, the results of this study were slightly different from the results of the present study. Indeed, on cyclists, HIDIT did not significantly increase T_lim_, but the average ${\dot{\text{V}}\text{O}}_{{2}}$ during the HIIT with long intervals was higher than HIIT with short intervals. In the present study, we wanted to verify that HIDIT was advantageous also in running performance, since the kinetics of ${\dot{\text{V}}\text{O}}_{{2}}$ are not the same in running and cycling (Hill et al. 2003). Although in recent years several studies have tried to propose strategies to increase the time close to ${\dot{\text{V}}\text{O}}_{{{\text{2max}}}}$ during HIIT, researchers have focused mainly on the fast start strategy to exploit the priming effect (Billat et al. 2013; De Aguiar et al. 2013; Lisbôa et al. 2015; Bossi et al. 2019; Rønnestad et al. 2019, 2021; Beltrami et al. 2021). Additionally, most of the studies were performed on cycle ergometers. On the other hand, the studies by Rønnestad et al. were conducted on cross-country skiers and confirmed that a fast-starting strategy can increase the average ${\dot{\text{V}}\text{O}}_{{2}}$ (Rønnestad et al. 2019, 2021) and the time above 90% of the ${\dot{\text{V}}\text{O}}_{{{\text{2max}}}}$ (Rønnestad et al. 2021) compared to a traditional HIIT session. Moreover, the study by (Beltrami et al. 2021) is particularly interesting because the authors compared a fast start protocol and a traditional HIIT protocol in runners and cyclists. The authors showed that only in cycling performance the average V̇O_2_ and the time above 90% of the ${\dot{\text{V}}\text{O}}_{{{\text{2max}}}}$ were greater. Conversely, when subjects were asked to perform the same protocol by running, the ${\dot{\text{V}}\text{O}}_{{2}}$ kinetics were not different between the two HIIT protocols. Although the tests by Beltrami and colleagues did not lead to exhaustion, there seem to be some similarities between the fast start strategy and HIDIT during running. In fact, our data show that there is no difference in the average ${\dot{\text{V}}\text{O}}_{{2}}$ between HIDIT and traditional protocols. Therefore, for the same duration, the two protocols would not have shown differences over time above 90% of ${\dot{\text{V}}\text{O}}_{{{\text{2max}}}}$.

### Practical applications

In adult active males, training protocols starting with long intervals followed by short intervals could be useful to accumulate time close to ${\dot{\text{V}}\text{O}}_{{{\text{2max}}}}$ and consequently improve it. HIDIT would not seem physiologically more fatiguing than other classical protocols. Thus, it could be used in a general preparation phase in the same way as traditional HIIT protocols. Women were not included among the participants, this precluded us from verifying whether HIDIT can also be successfully applied to women's training.

## Conclusions

In conclusion, during the HIIT trials performed for this study, postponing the first recovery interval below CV contributed to increasing T_lim_. Furthermore, active males achieved longer time above 90% of ${\dot{\text{V}}\text{O}}_{{{\text{2max}}}}$ and longer T_lim_ by performing a HIDIT running protocol than HIIT protocols with long and short intervals, despite having similar values of BLC and RPE at the end of the trial.

## Abbreviations

BLC: Blood lactate concentration
CP: Critical power
CR-10: Validated scale of perceived exertion
CV: Critical velocity
CV_6_: Velocity leading to exhaustion in 6 min
D’: The maximum distance that may be covered beyond CV
ES: Effect size
HIDIT: Decreasing intervals HIIT (combining high intensity from 3 min to 30 s and low intensity from 2 min to 20 s)
HIIT: High-intensity interval training
HR: Heart rate
LI_HIIT_: Long intervals HIIT (3 min high–2 s low intensity)
SI_HIIT_: Short intervals HIIT (30 s high–20 s low intensity)
Rec_30s_: Test consisting in a bout of exercise of 30 s followed by a recovery phase and a subsequent bout to exhaustion
Rec_3min_: Test consisting in a bout of exercise of 3 min followed by a recovery phase and a subsequent bout to exhaustion
Rec_Tlim_: Test consisting in a bout of exercise to exhaustion followed by a recovery phase and another bout to exhaustion
RPE: Rate of perceived exertion
T_lim_: Time to exhaustion
T90% ${\dot{\text{V}}\text{O}}_{{{\text{2max}}}}$: Time elapsed above 90% of V̇O_2max_
$${\dot{\text{V}}\text{CO}}_{{2}}$$: CO_2_ output
$${\dot{\text{V}}\text{O}}_{{2}}$$: Pulmonary O_2_ uptake
${\dot{\text{V}}\text{O}}_{{{\text{2max}}}}$: Maximal aerobic power
${\text{v}}{\dot{\text{V}}\text{O}}_{{{\text{2max}}}}$: Running speed occurred at the maximal aerobic power
W’: Amount of work that can be done during exercise above the CP
